# Book Review

**Published:** 1949-07

**Authors:** 


### Reviews of Books

**Elementary Atlas of Cardiography.** By H. Wallace-Jones, M.D., M.Sc., F.R.C.P., E. Noble Chamberlain, M.D., M.Sc., F.R.C.P. and E. L. Rubin, M.D., F.F.R., D.M.R.E. Third Edition. Pp. 108. Illustrated. Bristol: John Wright & Sons Ltd. 1948. Price 12s. 6d.—This little book of just over a hundred pages gives the student a sound elementary introduction to the difficult subject of electrocardiography and cardiac radiology. The illustrations throughout are good. The book fulfils its function admirably and can be thoroughly recommended.

**Physical Signs in Clinical Surgery, Pts. I–IV** (complete). By Hamilton Bailey, F.R.C.S., F.A.C.S., F.I.C.S., F.R.S.E. Eleventh Edition. Pp. xiii, 426. Illustrated. Bristol: John Wright & Sons Ltd. 1948-49. Price 8s. 6d. per part.—Shortages of bookbinders and of colour-block makers caused the author to lose patience and to decide to issue this edition in paper covers, and in four parts. As in former editions, the illustrations, 657 in number and mostly coloured, constitute by themselves a liberal education in surgery for student, practitioner and surgeon alike. The text also is very good and thoroughly up to date. Some of the information would be hard to come by elsewhere, and gives evidence of wide reading.

**Cardiovascular Disease.** By Terence East, D.M., F.R.C.P. Third Edition. Pp. 218. Illustrated. London: H. K. Lewis & Co. Ltd. 1949. Price 15s.—The fact that a third edition of this valuable little book has been needed in little more than three years is ample proof of its well-deserved popularity. It provides in simple and clear style a very comprehensive review of cardiovascular disease for the general practitioner. It is surprising how much information it has been possible to convey in a comparatively small book by eliminating all but essentials. Throughout, the physiological approach to the problem is maintained and the reader is shown how to approach cardiovascular problems from first principles. In this new edition some additions have been made and the whole has been brought up to date. This is a book which can be thoroughly recommended as a sound guide to cardiovascular diseases, both for the practitioner and student.

**Medical Annual, 1948.** Edited by Sir Henry Tidy, K.B.E., D.M., F.R.C.P. and A. Rendle Short, M.D., B.S., B.Sc., F.R.C.S. Pp. 414. Illustrated. Bristol: John Wright & Sons Ltd. 1948. Price 25s.—The sixty-sixth volume of the *Medical Annual* maintains the standard for which it has always been noted. It is unquestionably the best annual of recent advances in medicine and surgery that is published anywhere in the world. Everything concerned with recent progress from abdominal surgery to vitamins and nutrition is discussed in an interesting manner by recognized masters in the profession. It should be found in the library of every up-to-date medical practitioner.

**Clinical Endocrinology.** By A. P. CAWADIAS, O.B.E., M.D., F.R.C.P., Pp. iv., 362. Illustrated. London: Frederick Muller Ltd. 1947. Price 42s.—Instead of the usual approach from the point of view of physiology and pathology, Dr. Cawadias, as stated in his preface, here discusses endocrinology from the clinical point of view and he sets out for the practitioner an interesting and up-to-date exposition of diseases of the ductless glands. As would be expected, the work is accurate and shows evidence of wide and mature knowledge, and perhaps the only criticism would be that occasionally rather cumbersome phrases are interjected. Both for the student and the practitioner this book will prove a very helpful companion in his studies.

**The Clinical Apprentice.** By John M. NAISH, M.D., M.R.C.P., and JOHN APLEY, M.D., M.R.C.P. Pp. xi, 200. Illustrated. Bristol: John Wright & Sons Ltd. 1948. Price 15s.—The writers state that "this book has been written expressly for students receiving their clinical training in medicine". After a general survey, the various systems of the body are then considered from the point of view of the clinical approach and a helpful concluding section considers the problem of the acute case. Coma, meningism, hyperpyrexia, dyspnœa and fits are briefly but helpfully discussed and in the next edition it would be helpful to say more about cyanosis. Generally speaking, the information is accurate and precise and only a few errors incidental to a first edition are found here and there. While admitting the importance of feeling the pulse, it is not correct to delude the student into thinking that he can estimate blood-pressure in that way to such an extent that he may gain for himself the reputation of being "a human sphygmomanometer". Sterterous breathing is by no means a necessary feature of deep coma. Such errors, however, detract but little from a book which can be said to be a helpful introduction for the student who is commencing his clinical career.

